# Long-term Monocyte Dysfunction after Sepsis in Humanized Mice Is Related to Persisted Activation of Macrophage-Colony Stimulation Factor (M-CSF) and Demethylation of PU.1, and It Can Be Reversed by Blocking M-CSF *In Vitro* or by Transplanting Naïve Autologous Stem Cells *In Vivo*

**DOI:** 10.3389/fimmu.2017.00401

**Published:** 2017-05-01

**Authors:** Natalia Lapko, Mateusz Zawadka, Jacek Polosak, George S. Worthen, Gwenn Danet-Desnoyers, Monika Puzianowska-Kuźnicka, Krzysztof Laudanski

**Affiliations:** ^1^2nd Department of Anesthesiology and Intensive Care, Medical University of Warsaw, Warsaw, Poland; ^2^Faculty of Medicine, Ivano-Frankivsk Medical Institute, Ivano-Frankivsk, Ukraine; ^3^Department of Human Epigenetics, Mossakowski Medical Research Centre, PAS, Warsaw, Poland; ^4^Department of Pediatrics, Children’s Hospital of Philadelphia, Philadelphia, PA, USA; ^5^Department of Medicine, University of Pennsylvania, Philadelphia, PA, USA; ^6^Department of Geriatrics and Gerontology, Medical Centre of Postgraduate Education, Warsaw, Poland; ^7^Department of Anesthesiology and Critical Care, University of Pennsylvania, Philadelphia, PA, USA

**Keywords:** monocyte, dendritic cells, stem cells, macrophage-colony stimulation factor, PU.1, epigenetic regulation, immunosuppression, sepsis

## Abstract

The duration of post-sepsis long-term immune suppression is poorly understood. Here, we focused on the role of monocytes (MO) as the pivotal cells for long-term regulation of post-sepsis milieu. Lost ability of MO to adapt is seen in several acute conditions, but it is unclear for how long MO aberrancy post-sepsis can persist. Interestingly, the positive feedback loop sustaining secretion of macrophage-colony stimulation factor (M-CSF) can persist even after resolution of sepsis and significantly alters performance of MO. Here, we investigated the activation of M-CSF, and it as critical regulator of PU.1 in mice surviving 28 days after sepsis. Our primary readout was the ability of MO to differentiate into dendritic cells (DCs; MO→iDC) *in vitro* since this is one of the critical processes regulating a successful transition from innate to acquired immunity. We utilized a survival modification of the cecal ligation and puncture (CLP) model of sepsis in humanized mice. Animals were sacrificed 28 days after CLP (t_CLP+28d_). Untouched (CONTR) or sham-operated (SHAM) animals served as controls. Some animals received rescue from stem cells originally used for grafting 2 weeks after CLP. We found profound decrease of MO→iDC in the humanized mice 28 days after sepsis, demonstrated by depressed expression of CD1a, CD83, and CD209, diminished production of IL-12p70, and depressed ability to stimulate T cells in mice after CLP as compared to SHAM or CONTR. *In vitro* defect in MO→iDC was accompanied by *in vivo* decrease of BDCA-3^+^ endogenous circulating DC. Interestingly, post-CLP MO had persistent activation of M-CSF pathway, shown by exaggerated secretion of M-CSF, activation of PU.1, and demethylation of *SPII*. Neutralization of the M-CSF *in vitro* reversed the post-CLP MO→iDC aberration. Furthermore, transplantation of naïve, autologous stem cell-derived MO restored CLP-deteriorated ability of MO to become DC, measured as recovery of CD1a expression, enhanced production of IL-12p70, and ability of IL-4 and GM-CSF MO to stimulate allogeneic T cells. Our results suggest the role of epigenetic mediated M-CSF aberration in mediating post-sepsis immune system recovery.

## Introduction

During sepsis, an early, innate response transforms into acquired immunity-driven processes followed by a resolution (1, 2). It was believed that restoration of the immune system is complete. However, some sepsis survivors continue to succumb to secondary septic episodes, resurgent organ dysfunction (kidney failure, cognitive decline, and others), latent infections, or malignancies for several years after the initial septic episode (3–5). This may imply that the first insult hampers the patient’s immune system to subsequent challenges (2, 6). The mechanisms of such long-term decline remain largely under investigated (5).

Numerous mechanisms are involved in the homeostasis of the immune system (1, 2, 6). Antigen-presenting cells (APCs), especially dendritic cells (DCs) and monocytes (MO), are pivotal. They are able to recognize pathogen patterns or intercept antigens, a critical step in eliminating bacterial infections, halting the increase of viral load, and eradicating certain neoplastic growths (2, 7, 8). MO and APC are also pivotal in the de-escalation of inflammation and in wound healing (9). In sepsis, the circulating peripheral blood MO population serves as the primary fast track for DC emergence, but emerging DCs may have diminished stimulatory capacity (2, 8, 10). In sepsis, MO predominantly differentiates into macrophage (MAC), but even small numbers of emerging DC represent significant regulatory capacity. Thus, in the optimal immune response, there is a balance between MO differentiation into either DC or MAC, facilitating optimal immune system function and homeostasis (2, 7, 8, 11). Conversely, diminished MO to DC differentiation (MO→iDC), and MAC predominance, has been shown to correlate with adverse clinical outcomes in sepsis and other clinical conditions short term (10, 12–17). However, it is not clear how long these abnormalities persist. Plausible mechanisms of sustained decline in MO→iDC in the aftermath of sepsis remain elusive as well. Macrophage-colony stimulating factor (M-CSF) is abundantly produced by leukocytes, especially during inflammation, affecting MO’s ability to become DC *in vitro*. Its secretion can be sustained by a positive feedback loop even when initial stimulus has resolved. Prolonged stimulation with M-CSF has been linked to negative outcomes in trauma, burn, heart failure, and surgical recovery.

Here, we hypothesize that sepsis results in prolonged aberration in the plasticity and function of the MO well beyond the acute phase of sepsis. Prior work led us to formulate a more focused hypothesis suggesting that the sustained production of M-CSF is potentially responsible by virtue of preventing an optimal emergence of regulatory DC, long after sepsis resolution (18–21). Hence, by performing an autologous transplant of naïve stem cell precursors or reducing prolonged overproduction of M-CSF, the MO’s ability to become DC can be restored. We utilized a survival modification of the cecal ligation and puncture (CLP) model of sepsis and focused on long-term recovery instead of the acute phase (1, 20, 22–24).

## Materials and Methods

### Humanized Mice

A permit for study was approved by the Institutional Animal Care and Use Committee at the University of Pennsylvania. Animals were care in accordance in the USDA Animal welfare Act and all efforts were made in order to minimize number of enrolled animals and their suffering. Sublethally irradiated (3 Gy/mouse) NSG mice were transplanted with human umbilical cord blood CD34^+^ cells (1–1.5 × 10^5^ cells/mouse, intravenous) as previously described (23). Human immune reconstitution was assessed using flow cytometry (peripheral blood %CD45^+^ = 63.8 ± 14.03, %CD3^+^ = 36.6 ± 10.5, %CD19^+^ = 22.9 ± 7.54, %CD33^++^ = 38.1 ± 10.58) and similar to other researchers (20, 23). No significant changes were noted post-CLP at 28 days (peripheral blood %CD45^+^ = 75.8 ± 31.82, %CD3^+^ = 31.8 ± 6.61, %CD19^+^ = 29.1 ± 8.01, %CD33^++^ = 36.1 ± 12.11) in small pilot run. Also, no difference in peripheral blood leukocytes was seen in post-CLP animals re-transplanted with naïve stem cells (peripheral blood %CD45^+^ = 76.2 ± 7.21, %CD3^+^ = 30.8 ± 7.53, %CD19^+^ = 17.4 ± 8.43, %CD33^++^ = 33.0 ± 14.17).

### CLP Model of Sepsis

The CLP model was derived from pre-existing literature and modified for survival (23–26). Briefly, an animal was rendered unconscious with inhalation anesthesia, and the suture was placed at cecal appendage. Two punctures were performed using a 25-gauge needle. The fascia and skin were closed, and 0.5 ml of 0.25% bupivacaine was injected along the suture line. In sham animals, all the above steps would be performed except ligation and perforation of the cecum. One milliliter of 0.9% NaCl was injected in a scarf to hydrate animals every 24 h for 5 days. Antibiotics (12.5 mg/kg metronidazole and 25 mg/kg ceftriaxone) were administered every 12 h for a total of 5 days. Post-procedure animals were assessed daily for mortality assessment. Access to water and food was *ad libidum*. Day/night cycle was maintained throughout. At 28 days after CLP, the animals were sacrificed. Technical and clinical details of this model are published in another manuscript (under review).

Some animals after CLP were reinjected intravenously with stem cells (2 × 10^5^ cell/animal) originally used to graft the animals 2 weeks after CLP or sham procedure.

### Generation of Immature DCs from MO

Blood was collected during exsanguination. Some of the peripheral blood leukocytes were subjected to flow cytometry in order to evaluate the frequency of CD3, CD19, CD33, CD45, and BDCA-3 positive cells. Remaining blood, spleen, and bone marrow were used for an independent separation of intact monocytes using the negative section with the use of magnetic beads as described before (17, 27). The purity of the cells exceeded 95% for CD33^(bright)^ and 80% for CD14^(+)^. Isolated MO were incubated in X-VIVO 10/15™ media with gentamycin and phenol red (Lonza, Cohasset, MN, USA), supplemented with human IL-4 (Peprotech, Rocky Hill, NJ, USA) at 500 IU/ml and human GM-CSF (Peprotech, Rocky Hill, NJ, USA) at 1,000 IU/ml at 37°C and 5% CO_2_. On day 3, 50% of the supernatant was collected and replenished with fresh media and cytokines. After 5 days, the supernatants and cells were collected using an ice-cold 10 mM EDTA buffer (Gibco, Grand Island, NY, USA), followed by a washout with PBS without Ca^2+^ or Mg^2+^ (Gibco, Grand Island, NY, USA) (27). In some experiments, rabbit polyclonal neutralizing antibody for M-CSF (FN M-CSF, 5 µg/ml) was added at the beginning of the culture. The dose was shown to fully neutralized M-CSF in prior study (17). An addition of non-specific IgG had no effect on MO function (17).

### Flow Cytometry

A total of 10^5^ cells were incubated with a flow cytometry media (PBS w/o Ca^2+^ or Mg^2+^ with 0.01% sodium azide and 1% fetal bovine serum) enriched with human TrueStainFcX™ (BioLegend, San Diego, CA, USA) for 15 min at 4°C in the dark. Cells were then incubated with the antibodies for 30 min at 4°C in the dark, with additional mixing at 15 min. Cells were washed twice in FACS media and re-suspended in 100 µl of 1% FlowFix (Polysciences, Warrington, PA, USA). The following antibodies were employed: CD1a (HI149; BioLegend, San Diego, CA, USA), CD14 (Tuk4; Invitrogen, Grand Island, NY, USA), CD83 (HB15e; BD, San Jose, CA, USA), CD115 (AFS98; BioLegend, San Diego, CA, USA), CD124 (G077F6; BioLegend, San Diego, CA, USA), and CD126 (UV4; BioLegend, San Diego, CA, USA).

The frequency of naturally occurring DC was analyzed with a BDCA enumeration kit (Myltenyi Biotec, Germany) (15, 28). Enumeration protocol takes into account leukocytes count in the blood.

Cells were analyzed with an LSR™ (BD, San Jose, CA, USA) or a FACSCalibur™ (BD, San Jose, CA, USA). Non-specific antibodies were used as a negative control. At least 10 × 10^4^ were collected. Data were expressed as a frequency of cells while mean fluorescent intensity (MFI) was a measure of receptor density. MFI is presented as relative to non-specific binding after exposing cells to non-specific antibodies.

### Mixed Lymphocyte Reaction (MLR)

A one-way MLR was employed (27). A total of 2 × 10^4^ harvested cells (IL-4 and GM-CSF differentiated MO) were added to 2 × 105 allogeneic T cells and incubated for 24 h. Twenty microliters of Alamar Blue (Life Technologies, Grand Island, NY, USA) were subsequently added. After 18 h of incubation, the absorbance at 570 nm (with a reference filter at 630 nm) was measured to assess T cell proliferation using the Opsys MR (Thermo Laboratories, Philadelphia, PA, USA) with Revelation software (Thermo Laboratories, Philadelphia, PA, USA) (27).

### Stimulation of IL-4&GM-CSF Differentiated MO

A total of 2 × 10^4^ IL-4 and GM-CSF stimulated myeloid cells were added to 200 µl of X-VIVO 10/15™ media with gentamycin (10 µg/ml) and phenol red (Lonza, Cohasset, MN, USA) supplemented with lipopolysaccharide (LPS) (Sigma Alrdich, St. Louis, ME, USA) (10 ng/ml) (27). After 24 h of incubation, 150 µl of supernatant was collected for cytokine analysis.

### Cytokine and Serum Marker Measurements

The level of cytokines (TNFα, IL-6, IL-12p70, and M-CSF) in supernatants were measured using a magnetic multiplex kit (Bio-Rad, Hercules, CA, USA) as per the manufacturer’s protocol and analyzed on the BioRad™ platform (Hercules, CA, USA).

### Epigenetic Analysis of PU.1/*SPI1* Methylation

In order to assess epigenetic regulation of PU.1, an analysis of methylation of *SPI1* was conducted. Using the CpG Island Finder program (http://dbcat.cgm.ntu.edu.tw), we analyzed 3 kb fragment of the *SPI1* gene encoding the PU.1 transcription factor. The analyzed fragment was located from 1.5 kb upstream to 1.5 kb downstream of the position corresponding to the first base of the major gene transcript (potential transcription start site). Within the 750-bp long fragment indicated as CpG island, a 375-bp fragment including potential transcription start sites of the major and two other gene transcripts, containing 17 CpG dinucleotides, was selected for further analysis. The methylation status of this fragment was analyzed using the OneStep qMethyl Kit (Zymo Research, Irvine, CA, USA) following the manufacturer’s protocol. The real-time PCR reaction was performed using LightCycler 480 II (Roche Diagnostics, Mannheim, Germany). The primers used were: forward 5′ATGTCACCCCAAGGGGACTA3′ and reverse 5′GGAAACCCTGACTTCCCACT3′. The PCR conditions were: initial denaturation for 10 min at 95°C, then 45 cycles of 30 s at 95°C, 30 s at 63°C, 30 s at 72°C, and then one melting curve cycle.

### Statistical Analysis

The study was designed as a cohort study of the ability of MO to become professional iDC under the influence of IL-4 and GM-CSF *in vitro* using several indices of this process (%CD1a^+^, production of IL-12p70, MLR reactivity). At least six independent animals were used from each study group for statistical contrast based on power analysis for primary outcome. The parametric nature of the data was confirmed by Levene’s and Shapiro–Wilk tests. Parametric data were presented as average (*X*) ± SD while non-parametric variables were denoted as median (M_e_) with 25–75% interquartile range (IQ). *t*-Tests and Wilcoxon matched pair tests were conducted depending on the characteristics of the data. ANOVA was conducted for multiple group comparisons with a Bonferroni test for *post hoc* analysis. The data were flagged as significant if the two-tailed hypothesis test was <0.05, unless otherwise specified. Statistica v8.0 (Statistica; Tulsa, OK, USA) was used.

## Results

### Ability of MO to Become DC Is Severely Depressed in Post-CLP Surviving Mice

**Figure 1:**
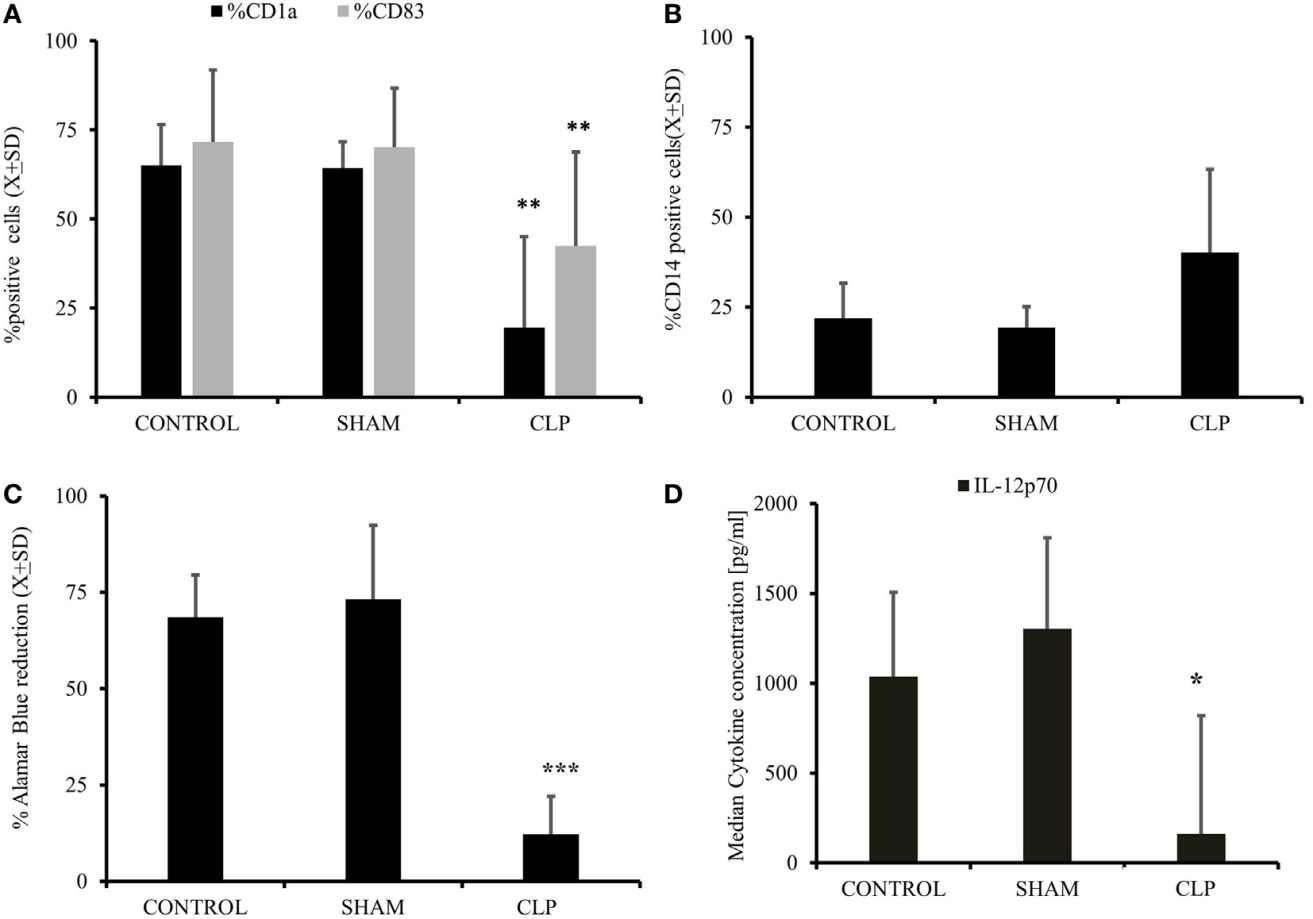
**Cecal ligation and puncture (CLP) results in long-term diminished ability of myeloid cells to become iDC *in vitro* after initial sepsis**. Isolated monocytes (MO) obtained from untouched (CONTR; *n* = 8), sham-operated (SHAM; *n* = 6), and surviving 28 days after CLP (CLP; *n* = 14) were stimulated with IL-4&GM-CSF *in vitro* for 5 days followed by harvest to assess the degree of MO to iDC differentiation. Flow cytometry was used to assess the emergence of dendritic cell (DC)-specific markers like CD1a and CD83 **(A)**. CD14, a marker for undifferentiated monocyte, and not present typical on the surface of DC was assessed as well **(B)**. This flow cytometric evidence of the effectiveness of MO to iDC differentiation process was accompanied measure of the IL-4&GM-CSF-stimulated MO cells to stimulate allogeneic T cells in mixed lymphocyte reaction. IL-4&GM-CSF differentiation MO from each studied group were incubated 1:100 ratio with the presence of Alamar Blue, reduction of which represents the level of stimulation and antigen-presenting cell proficiency **(C)**. We also incubated with IL-4&GM-CSF differentiation MO lipopolysaccharide to assess the supernatants level of IL-12p70, a critical cytokine for T cells stimulation **(D)**. Bars represent average of results (*X*) with SD. Data were analyzed by one-way ANOVA for multiple group comparisons with a Bonferroni test for *post hoc* analysis (*p < 0.05, **p < 0.01, ***p < 0.001).

In the first step, we assessed the ability of MO obtained from post-CLP survivors to become iDC under the influence of exogenous IL-4&GM-CSF (11, 17, 27). IL-4&GM-CSF-differentiated MO obtained from mice surviving sepsis had significantly lower expression of CD1a and CD83, two exclusive markers for monocyte-derived immature and mature cells, respectively (Figure 1A). Expression of monocyte marker, CD14, on the surface of IL-4&GM-CSF-differentiated MO appeared to be non-significantly different among the studied groups due to the non-parametric nature of the data (*p* = 0.42) (Figure 1B). Moreover, the expression of CD209, a molecule induced early in the process of MO differentiation into iDC, was depressed in the CLP group as compared to SHAM-operated (SHAM) (%CD209^+^_SHAM_ = 77.7 ± 15.9 vs %209^+^_CLP_ = 55.2 ± 21.8; *p* = 0.03) (29, 30). The ability of IL-4&GM-CSF-differentiated MO to stimulate allogeneic T cells in MLR was severely depressed in the post-CLP group (Figure 1C). Next, we focused on the expression of CD86, one of the critical co-receptor for the activation of T cells (10, 12, 27). Receptor density was not significantly elevated on the surface of IL-4&GM-CSF-differentiated MO at 28 days after surgery (M_e_[IQ_25–75%_]; MFI CD86_CONTROL_ = 290[272–308] vs CD86_SHAM_ = 381[347–424] vs CD86_CLP_ = 289[253–689]; *p* = 0.713). Finally, IL-4&GM-CSF-differentiated MO stimulated with LPS secreted significantly less IL-12p70 (Figure 1D), while production of M-CSF was increased by MO differentiated with IL-4&GM-CSF but only in animals surviving 28 days after CLP (*p* = 0.018) (Table 1).

**Table 1:** **Secretion of M-CSF in IL-4&GM-CSF differentiated MO was elevated in animals surviving sepsis**.

	CONTR	SHAM	Cecal ligation and puncture	*p*-Value
MO to iDC M-CSF (*X* ± SD) (pg/ml)	2,235 ± 222.9	1,966 ± 206.1	4,257 ± 1,282.3^a^^,^^b^	0.005
MO LPS M-CSF M_e_[IQ25–75%] [pg/ml]	463[135–585]	456[391–576]	5,351[3,161–5,928]^a^	0.002
MO LPS TNFα M_e_[IQ25–75%] (pg/ml)	8,895[7,723–9,486]	8,769[8,206–12,963]	5,093[4,933–6,296]	NS
MO mean fluorescent intensity (MFI) TLR4 (*X* ± SD)	1,578 ± 269.9	1,286 ± 359.1	1,249 ± 70.52	NS
MO%CD14 (*X* ± SD)	81.7 ± 7.26	79.8 ± 3.98	84.1 ± 2.81	NS
MO MFI CD14 (*X* ± SD)	3,760 ± 1,153.9	4,306 ± 592.2	4,348 ± 1,144.1	NS

*Concomitantly, macrophage-colony stimulation factor (M-CSF), but not TNFα, secretion by MO in response to lipopolysaccharide (LPS) was elevated as well in the same group. However, expression of receptor pivotal for LPS recognition (TLR4 and CD14) was not different across studies group*.

*MO, monocyte; DC, dendritic cells; M_e_, median; IQ, interquartile intervals*.

*^a^Denotes significance vs CONTR*.

*^b^Denotes significance vs SHAM-operated (SHAM)*.

### The Frequency of Only BDCA-3^+^ Positive Endogenous Circulating DC Is Depressed after CLP

We assessed the frequency of naturally occurring DC using specific markers (15, 28). The frequency of BDCA-3^+^ was significantly lower in CLP survivors as compared to sham-operated animals at 28 days after surgery (%BDCA-$3_{\text{sham}}^{+}$ = 0.078 ± 0.012 vs %BDCA-$3_{\text{CLP}}^{+}$ = 0.055 ± 0.014; *p* = 0.03), while other types of endogenous DC remained the same or tended to slightly increase (%BDCA-$2_{\text{sham}}^{+}$ = 0.1 ± 0.2 vs %BDCA-$2_{\text{CLP}}^{+}$ = 0.12 ± 0.15; *p* = ns) (%BDCA-$4_{\text{sham}}^{+}$ = 0.2 ± 0.18 vs %BDCA-$4_{\text{CLP}}^{+}$ = 0.17 ± 0.23; *p* = ns). When recalculated for absolute number of BDCA-positive cells, we detected similar tendencies since there was no difference in absolute number of leukocytes per milliliter of blood (data not shown).

### Decreased Ability of MO to Become iDC under the Influence of IL-4&GM-CSF Is Accompanied by Increased Expression M-CSFR

**Figure 2:**
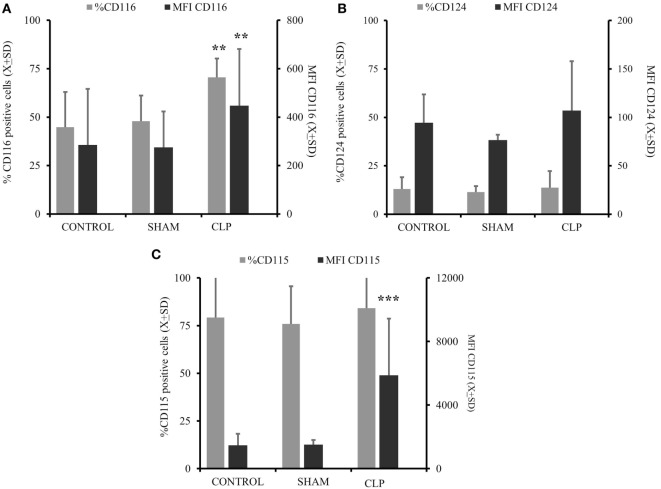
**There is significant predominance of GM-CSF_R_ and M-CSF_R_ on the circulating monocytes cells post cecal ligation and puncture (CLP)**. Monocytes (MO) obtained from all three studied groups [CONTR *n* = 10, SHAM-operated (SHAM) *n* = 6, CLP *n* = 8] were incubated with specific surface antibodies against the receptors supporting differentiation of MO into dendritic cell (DC) (CD116 combined with CD124) **(A,B)** and receptors cytokines preventing MO to iDC differentiation like CD115 **(C)** or CD116 *alone* **(A)**. The flow cytometry was conducted on at least 10,000 cells using FS/SS scatters to exclude clumps and dead cells. Surface expression is present as a percent of positive cells (%) and mean fluorescent intensitiy (MFI) on the gates cells. Bars represent average of results (*X*) with SD. Data were analyzed by one-way ANOVA for multiple group comparisons with a Bonferroni test for *post hoc* analysis (*p < 0.05, **p < 0.01, ***p < 0.001).

In the next step, we assessed the ability of MO to respond to differentiating cytokines *in vitro* (7, 10, 11, 17, 27). The expression of GM-CSF_R_ (CD116) was elevated in the CLP group in terms of frequency of positive cells (%) (*p* = 0.0019) and receptor density (MFI) (*p* = 0.0013) (Figure 2A). IL-4_R_(CD124) expression was not different between the three studied groups (Figure 2B). When we analyzed the expression of receptors for cytokines inhibitory for the MO→iDC process, we found no increase in IL-6_R_ (CD126; %CD12$6_{\text{sham}}^{+}$ = 81.38 ± 9.03 vs %CD12$6_{\text{CLP}}^{+}$ = 82.8 ± 9.93; *p* = ns) (MFI CD12$6_{\text{sham}}^{+}$ = 1,116.2 ± 748.58 vs MFI CD12$6_{\text{CLP}}^{+}$ = 1,171.6 ± 812.58; *p* = ns). However, the expression of M-CSF_R_ (CD115) was significantly elevated in terms of average receptor density (*p* < 0.000051) (Figure 2C).

### Neutralization of Elevated Production of M-CSF *In Vitro* Corrects MO→iDC Blockade in MO Obtained from Humanized Mice Surviving CLP

Analysis of the cytokine profile in the supernatant of MO differentiated with IL-4 and GM-CSF revealed a significant elevation in production of M-CSF and TNFα but only in the cells obtained from the animals surviving CLP (Table 1). Similarly, when we analyzed the cytokine production by circulating MO stimulated with LPS *in vitro*, we found elevated levels of M-CSF but only in animals surviving sepsis (Table 1). The secretion of TNFα was not significantly changed in post-CLP animals as compared to SHAM. The elevated production of M-CSF was not related to increased expression of the toll-like receptor 4 or CD14, two critical components in LPS response (Table 1).

**Figure 3:**
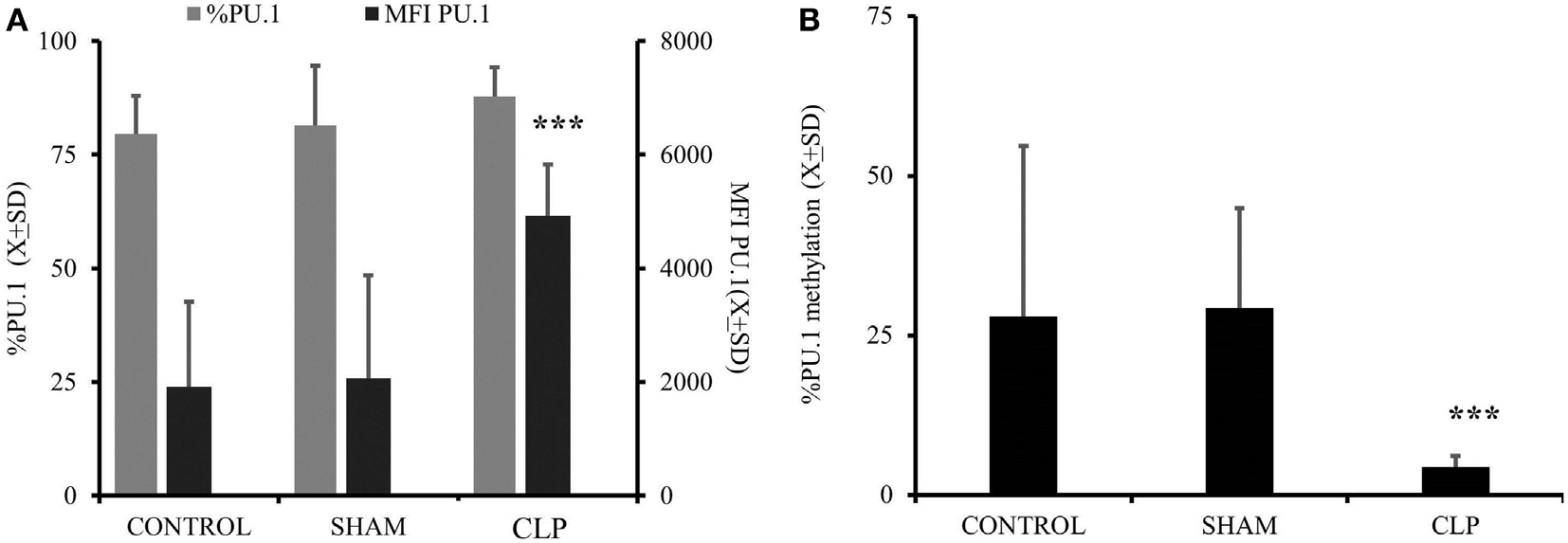
**Activation of macrophage-colony stimulation factor (M-CSF) was elevated in animals 28 days after cecal ligation and puncture (CLP) due to increased promotor activity and concomitant with demethylation of the promotor**. Monocytes (MO) obtained from bone marrow were collected from all three studied groups (CONTR; *n* = 6, SHAM; *n* = 6, CLP; *n* = 8). Dye tagged anti PU.1 antibody was incubated with permeabilized cells for 1 h and subjected for flow cytometry as described before. Expression of the PU.1 was showed as a difference in mean fluorescent intensity as compared to cells incubated with non-specific controlled dye tagged antibody in addition to percentage of positive cells (%) **(A)**. A 3-kb fragment of the *SPI1* gene encoding the PU.1 transcription factor was analyzed to measure the degree of the methylation using the same donors as for PU.1 measurements **(B)**. Bars represent average of results (*X*) with SD. Data were analyzed by one-way ANOVA for multiple group comparisons with a Bonferroni test for *post hoc* analysis (*p < 0.05, **p < 0.01, ***p < 0.001).

**Figure 4:**
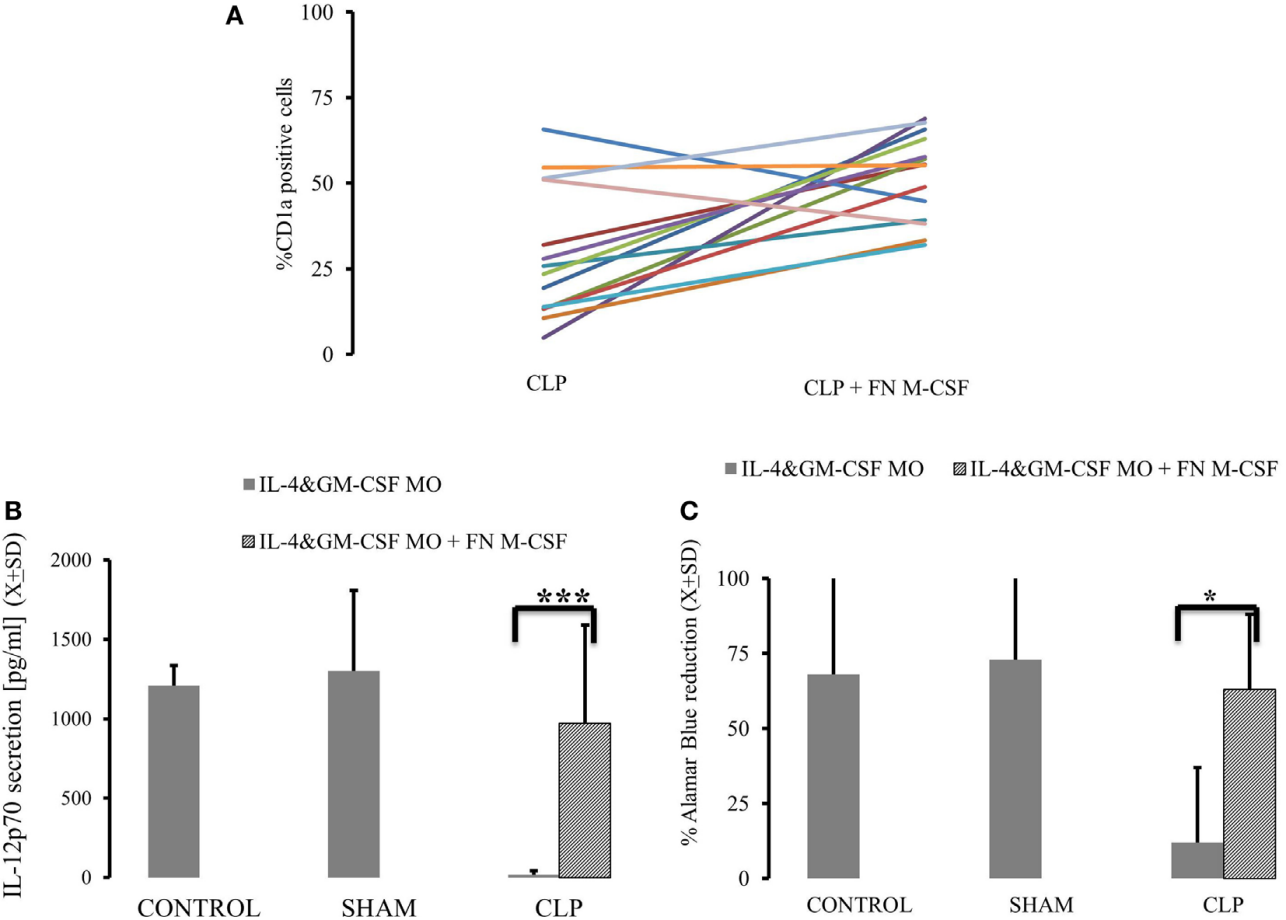
**Neutralizing macrophage-colony stimulation factor (M-CSF) results in restoration of monocytes’ (MO) ability to become dendritic cell (DC) in animals surviving cecal ligation and puncture (CLP)**. MO obtained from animals control animals (CONTR; *n* = 8), sham-operated (SHAM *n* = 6), and surviving CLP (CLP; *n* = 14) were incubated with IL-4&GM-CSF alone or with the presence of the antibody neutralizing M-CSF (FN M-CSF; 5 mg/ml). We presented expression of CD1a on the IL-4&GM-CSF alone and IL-4&GM-CSF + FN-M-CSF-enriched cultures from each single experiment for parallel comparisons **(A)**. We also measured recovery of the production of IL-12p70 **(B)** after lipopolysaccharide challenge in IL-4&GM-CSF alone and after adding FN M-CSF. Concomitantly, we measured ability of differentiated MO to stimulate T cells in mixed lymphocyte reaction showed as a reduction of Alamar Blue **(C)**. Solid gray bar represent experiment done with IL-4&GM-CSF alone. Oblique zebra bars represent experiments where FN-MC-CSF was supplemented. Bars represent average of results (*X*) with SD. Data were analyzed by *t*-test for parallel group comparisons (*p < 0.05, **p < 0.01, ***p < 0.001).

Considering that both expression of M-CSF_R_(CD115) and secretion of M-CSF are elevated up to 28 days after CLP, we focused on the M-CSF pathway as a potential culprit for depressed MO→DC (19, 20, 31, 32). Increased secretion in production of M-CSF was accompanied by increased activity of PU.1, a critical component in M-CSF induction (Figure 3A) (*p* = 0.000183) (18, 19, 33, 34). Furthermore, we found that there was a significant decrease in the methylation of the *SPI1* promoter fragment flanking the potential transcription start site of the main PU.1 transcript and two other transcripts in the CLP animals (Figure 3B) (*p* = 0.000021). Finally, we added a neutralizing antibody for M-CSF (FN M-CSF) to IL-4&GM-CSF-differentiated MO obtained from CLP animals and SHAM. We observe at least partial restoration of MO→iDC differentiation demonstrated by acquisition of DC markers (Figure 4A). We also observed recovery of the IL-12p70 production (*p* = 0.0008) if the MO obtained from CLP animals were differentiated in the presence of FN-M-CSF (Figure 4B). Concomitantly, the ability of MO to become effective allogeneic T cell stimulators was restored if post-CLP MO were incubated with FN M-CSF (Figure 4C) (*p* = 0.044). In contrast, FN-M-CSF was ineffective in further boosting CD1a level on IL-4&GM-CSF differentiation MO obtained from SHAM (data not shown).

### Autogeneic Transplant of Naïve Stem Cells Restores MO Potential to Become DC *In Vitro* and Population of Endogenous DC

**Figure 5:**
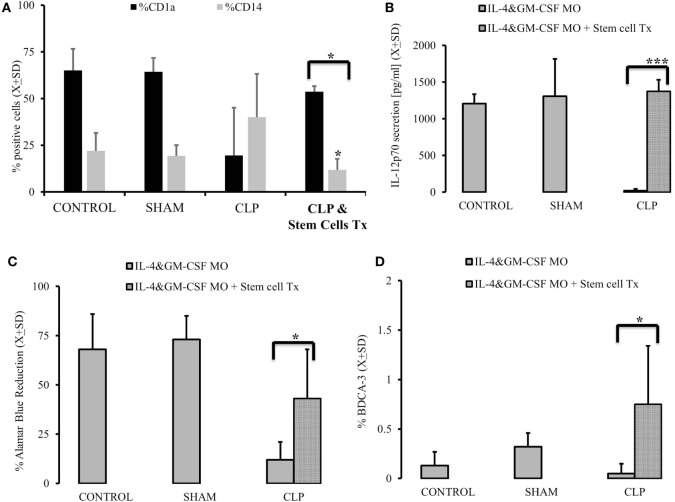
**Autogenous stem cells transplant restores the ability of monocytes (MO) to become dendritic cells (DCs) *in vitro* in cecal ligation and puncture (CLP) survivors**. MO obtained from animals control (CONTR; *n* = 8), sham-operated (SHAM *n* = 6), surviving CLP (CLP; *n* = 14), and CLP survivors receiving 2 × 10^5^ autogeneic stem cells (CLP&Tx; *n* = 7) 2 week after initial CLP. These animals were sacrificed 2 weeks later. Their MO isolated and subjected to IL-4&GM-CSF differentiated conditions using DC marked (CD1a) and MO marker (CD14) **(A)**. We also measured recovery of the production of IL-12p70 **(B)** after stem cell transplant vs CLP alone animals. Concomitantly we measured ability of differentiated MO to stimulate T cells in mixed lymphocyte reaction showed as a reduction of Alamar Blue **(C)**. Recovery of endogenous DBCA(+) DC was abserved as well **(D)**. Solid gray bar represent experiment done with cells obtained with CLP alone. Dotted bars represent experiments where 2 × 10^5^ autogeneic stem cells were conducted. Bars represent average of results (*X*) with SD. Data were analyzed by *t*-test for parallel group comparisons (*p < 0.05, **p < 0.01, ***p < 0.001).

In the last set of experiments, we injected the mice 2 weeks after CLP with the naïve stem cells originally used to graft the animals. When the animals were sacrificed 28 days after initial insult, we observed a partial restoration of MO’s ability to become DC, as evidenced by the increased expression of CD1a (*p* = 0.0058) on the surface of IL-4&GM-CSF-stimulated myeloid cells, while the expression of a monocyte/macrophage marker, CD14, was diminished (Figure 5A). Also, the ability of post-CLP IL-4&GM-CSF-differentiated MO was restored to almost pre-CLP levels when production of IL-12p70 (*p* = 0.000023) and ability of T cells to stimulate was assessed (Figures 5B,C) (*p* = 0.036). We also assessed the frequency of endogenous DC and found that the frequency of BDCA-3^+^ cells was significantly higher than in animals after CLP (Figure 5D) (*p* = 0.0022).

Interestingly, animals after stem cells transplant had secretion of M-CSF in response to LPS and comparable CONTR animals (CONTR_M-CSF after LPS_ = 406.1 ± 134.7 vs CLP_M-CSF after LPS_ = 4,319.3 ± 2,042.4; CLP + Stem Cell Tx_M-CSF after LPS_ = 1,118.1 ± 93.4; *p* = 0.0001). The same trend was seen during *in vitro* IL-4&GM-GM-CSF differentiation in respect to M-CSF secretion (CONTR_M-CSF after IL-4&GM-CSF_ = 2,235.2 ± 222.91 vs CLP_M-CSF after IL-4&GM-CSF_ = 4,062.3 ± 844.4; CLP + Stem Cell Tx_M-CSF after IL-4&GM-CSF_ = 1,930.1 ± 586.9; *p* = 0.0068). Methylation of PU.1 also partially normalized (CONTR_methylation of SPII_ = 27.9 ± 26.27 vs CLP_methylation of SPII_ = 4.4 ± 1.79; CLP + Stem Cell Tx_methylation of SPII_ = 12.3 ± 4.06; *p* = 0.0002).

## Discussion

In our study, we showed that myeloid cells 28 days post-sepsis have a diminished capacity to become DC *in vitro*. This *in vitro* defect was paralleled by sustained diminished frequency of endogenous circulating BDCA-3^(+)^ DC *in vivo*. An elevated level of M-CSF production in response to LPS as well as during differentiation of MO into DC was accompanied by upregulation in PU.1 and demethylation of *SPI1*. Finally, the diminished capacity of MO to become DC was reversed by neutralization of M-CSF *in vitro* or by transplanting autogenic, naïve stem cells *in vivo*. Our study provides an explanation as to how the immunological milieu may be altered by long-term sepsis leading to the emergence of comorbidities in some individuals (1, 2, 6, 26).

Here, we utilized a model of sepsis with a single discrete insult treated in similar way as in a typical clinical scenario and sacrificed 28 days after initial insult. Several studies showed depressed ability of MO to the emergence into DC after sepsis and other severe insult but these studies focused on acute phase or the individuals were exposed to multiple insults (10, 14, 17, 31, 35, 36). Differentiation of MO with IL-4&GM-CSF yields DC through a well-established protocol that mimics naturally occurring processes (11, 17, 27). Alternatively, MO can differentiate into CD1a^−^ population of DC with immunosuppressive properties. These alternatively activated DC emerged from CD16^(high)^ MO population but no increase in frequency of CD16^+^/CD33^+^ leukocytes were observed (data not shown). The preservation of CD14, a markers not present on the surface of any DC, suggest that MO obtained from humanized mice are MO arrested in a pre-DC process and are unable to acquire antigen-presenting features (37). This *in vitro* MO→DC differentiation defect was accompanied by diminished expression of BDCA-3(+) endogenous circulating DC. In contrast to tissue-residing DC (e.g., plasmacytoid, Langerhans), circulating DC are critical for several immune functions. Interestingly, we observed a diminished frequency of BDCA-3 that are pivotal for T cell stimulation and resemble the mouse equivalent of MO-derived DC (28, 38). Frequencies of BDCA-2 and BDCA-4, representing plasmocytoid and lymphoid were unchanged, suggesting that myeloid lineage may be predominantly affected post-sepsis (28, 39, 40).

Elevated expression of M-CSF_R_ on the surface of circulating MO suggests enhanced sensitivity to signals that differentiate them into macrophages. Predominance of macrophages has been reported up to 4 weeks after sepsis or critical care events but not in humanized mice (12, 17, 21, 41–43). Furthermore, we noticed that M-CSF production is elevated under resting and post-LPS stimulating conditions. M-CSF is a pivotal cytokine for the emergence of monocytes from bone marrow as well as their function during circulation and in peripheral tissues (18, 20, 21, 32–34, 44). This may suggest that monocytes are of pre-determined macrophage characteristics, as has been suggested before (1, 3, 7, 9, 15). However, similar expression of other macrophage markers across CLP and CONTR suggests that circulating MO (paper in publication) are “hyper-secretors” (8, 18, 44) of M-CSF not just macrophages secreting increase amount of M-CSF because of being macrophages. Furthermore, the unaltered expression of TLR4 receptor on the surface of post-CLP MO seems to negate the emergence of LPS tolerance and related M-CSF production (45). The increase in M-CSF secretion is coupled with elevated expression of its own receptor (CD115/M-CSFR), suggesting a reinforcing positive loop preventing MO from becoming DC and pushing their differentiation into “arrested” or alternatively activated monocytes (18, 21, 33, 41, 44). This effect of M-CSF can be neutralized thus reversing the block of MO→DC *in vitro* differentiation but only in CLP animals. Interestingly, the availability of commercial available neutralizing antibody for M-CSF opens up the possibility of therapeutic options (21). However, it is likely that M-CSF effects are modulated *via* paracrine fashion and exaggerated presence of the cytokine in the microenvironment supports the blocking of MO differentiation block. In our preliminary study, we measured the levels of M-CSF in the serum and we found that it was not being dramatically different among the studied groups. We also incubated MO with serum obtained from mice surviving sepsis but no significant biological effect could be appreciated (pilot data). These strongly suggested the paracrine effect of M-CSF and observed alteration. One has to keep in mind that M-CSF is critical for MO emergence from bone marrow and their survival in the circulation. Therefore, the techniques almost totally abrogating its production result in increased MO apoptosis or suboptimal function (31, 42). In contrast, neutralizing antibody allowed for some “background” effect of M-CSF instead of totally suppressing it. The concentration of FN M-CSF Ab was similar to use by others and resulted in undetectable level of M-CSF in supernatants. However, neutralizing antibodies are much less effective in paracrine neutralization. Our prior results, in which we demonstrated the recovery effect of FN M-CSF only in the patients with depressed (as compared to baseline) MO to iDC level, suggest that there is certain level above which the effect of M-CSF is inhibitory (17, 46). This observation is well aligned with the most understanding role of M-CSF as a trophic factor for MO survival (18, 19). FN M-CSF neutralizes a substantial part of an exaggerated secretion of M-CSF but preserves its beneficial effect on MO survival thus restoring pre-CLP balance (6, 47).

Persistent secretion of M-CSF has been observed in trauma patients, but the mechanism of sustained activation of M-CSF has not been elucidated. M-CSF can be sustained *via* several positive feedback mechanisms that are potentially self-sustaining even after the initial insult is gone (19, 32, 34). Here, we showed that expression of PU.1, a principal promoter of M-CSF secretion, is elevated after insult and persists for 28 days, even in recovering animals (33, 34). Interestingly, demethylation of the PU.1-encoding *SPI1* promoter was observed as well (48, 49). This epigenetic change can sustain the secretion of M-CSF, because *SPI1* expression is no longer inhibited thus providing a sustained epigenetic mechanism for M-CSF secretion. In a definitive experiment, we would try to manipulate with methylation of *SPI1* but none of the currently compounds is highly specific. One also has to keep in mind that M-CSF is critical for MO function and suppressing its levels with can result in MO loss. Therefore, using transfection experiments to affect activity of PU.1 in primary MO may be difficult due to the high incidence of cells necrosis.

Prolonged secretion of M-CSF can be triggered by sepsis but it is very likely sustained at the level of progenitors since the half-life of monocyte is 4–7 days. Also, the diminished frequency of DBCA-3(+) cells suggests bone marrow defect. Elevated level of PU.1, a critical factor in regulation of not only M-CSF but also other inflammatory and regulatory pathways, suggests that M-CSF can be one of the plausible effects of CLP (34, 49–51). Also, it has been shown that aberration in stem cells or, the mesenchymal supportive network of bone marrow, can prolong post-septic phenomenon on immune-aberrancy (47, 52, 53). Obtaining the cells before the septic insult and re-introducing them later would prevent exposure to the noxious cocktail of inflammatory mediators that affect the remaining circulating leukocytes and prime them for negative response (1, 47, 54). We showed that autogenetic transplant has a sustained and positive effect on reversing post-septic MO aberration. Concomitantly, the production of M-CSF and methylation of *SPI1* started to return to pre-CLP levels. Furthermore, we also observed the restoration of the endogenous DC population suggesting that other cells than MO are also affected in post-sepsis environment (17, 35, 36). The mechanism by which allogeneic transplant of stem cells restores leukocyte populations to pre-CLP levels is elusive. It takes 3 months for irradiated animals to rebuild their immune system (23). Naïve stem cells re-introduced into mice with an already matured human immune system might have potentially more favorable conditions but further study is necessary to establish the fate of these naïve stem cells. Since banking of cord blood is becoming more and more popular the restoration of immunity to pre-sepsis status using cord blood stem cells is plausible.

Our study has several limitations. We used the humanized mice and CLP in order to approximate the trajectory observed in patient with sepsis. However, humanized mice are *de facto* a xenotransplant model (22). It is unclear to what extent the response of the human leukocyte is adequate in mice environment considering the different functioning of critical immunological organs like bone marrow or the spleen. Furthermore, it is unclear how much HLA mismatch, lack of neuroendocrine influences, and unfavorable bone marrow environment affect the response of the immune system to CLP. A mismatch between the bone marrow supporting structures in the mice and human stem cells may affect the immune system response to CLP in creating post-septic equilibrium (23, 24, 32). Furthermore, bone marrow response to sepsis relies on the mesenchymal cells providing an optimal environment for differentiating leukocytes. In humanized animals, the influence of mesenchymal cells is somewhat limited considering species mismatch. Several other mechanisms are involved in the regulation of M-CSF, including miRNA, non-coding parts of DNA, and p38/Erk activation (50, 51, 55–57). Considering the limited amount of biological material, we decided not to focus on them considering that none of these mechanisms can create positive, self-sustained feedback loops. Also, the role of PU.1 on MO fate and their response is well studied on the level of bone marrow progenitors but its role in peripheral MO is much less well known. In our model, we studied only survivors of the CLP. We have no insight into M-CSF/PU.1/*SPI1* activation in animals which died during the experiment before sacrifice. One can only speculate if the loss of MO’s ability to become DC was more profound in these animals since this has not been studied. Our experiment do not proof definitively that M-CSF is the underlying culprit of diminished MO→DC. Elevated expression of M-CSF can be one of the long-term perturbations of CLP (1, 47). However, scarcity of the biological material prevented us from pursuing more sophisticated experiments. Also, we did not conduct genome-wide studies in order to provide insight into discovery of other pathways involved in long-term post-CLP ability of MO to become DC (7, 10, 31, 47). Although our model resembles a clinical trajectory than nature of the xenotransplant, animals made this model an approximation of sepsis.

Our system shows an interruption of the M-CSF pathway restores MO→DC *in vitro* while the introduction of naïve autogenic stem cells results in not only restoration of MO→DC but also SPII and M-CSF activity. Though we were not able to directly manipulate epigenetic regulation of M-CSF and PU.1 both experiment shows strong corroborating evidence for the role of sustained M-CSF secretion in post-sepsis immunosuppression as one of the potential consequences of sepsis.

## Conclusion

According to the common understanding, a singular insult to the immune system should result in temporal upset followed by recovery of the pre-insult balance. However, a new immuno-stasis can emerge after any acute or chronic condition (5, 6, 58). Our research supports the general idea that sepsis induces long-term changes after the initial episode. Our study showed an epigenetic demethylation in of PU.1 accompanied sustained influence of M-CSF is present in survivors of sepsis for 28 days after the initial insult and after resolution of acute phase of sepsis. Therefore, circulating MO, and bone marrow progenitors, are predominantly sensitive to cytokine signal prohibiting their differentiation into DC and induce tolergoenic M-CSF phenotype in the aftermath of sepsis. This is the first report of epigenetic mediated chronic changes in sepsis survivors. The post-sepsis diminished MO→DC is reversed *in vitro* by neutralizing M-CSF, or *in vivo* by re-transplanting post-CLP mice with autogeneic, naïve stem cells opening two very exciting therapeutic options. Though, we could not definitively show that M-CSF is primary and pivotal culprit of post-CLP changes, our research strongly suggest that restoring M-CSF/PU.1 axis recovers some features of immune system to pre-CLP level.

## Ethics Statement

The Animal Welfare Board at the University of Pennsylvania (Philadelphia, PA, USA) approved this study.

## Author Contributions

KL—study design, experiment, data collection, data analysis, and manuscript preparation. MZ—experiment, data collection, data analysis, and manuscript preparation. NL—data collection, analysis, and manuscript preparation. GW—study design, data analysis, and manuscript preparation. GD-D—study design, experiment, data collection, and manuscript preparation. JP—experiment, data analysis, and manuscript preparation. MP-K—experiment design, data analysis, and manuscript preparation.

## Conflict of Interest Statement

The authors declare that the research was conducted in the absence of any commercial or financial relationships that could be construed as a potential conflict of interest.
